# Microglia and Microglia-Like Cells: Similar but Different

**DOI:** 10.3389/fncel.2022.816439

**Published:** 2022-02-07

**Authors:** Miguel A. Cuadros, M. Rosario Sepulveda, David Martin-Oliva, José L. Marín-Teva, Veronika E. Neubrand

**Affiliations:** Department of Cell Biology, Faculty of Science, University of Granada, Granada, Spain

**Keywords:** microglia, microglia-like cells, yolk sac, bone marrow, hematopoietic stem cells

## Abstract

Microglia are the tissue-resident macrophages of the central nervous parenchyma. In mammals, microglia are thought to originate from yolk sac precursors and posteriorly maintained through the entire life of the organism. However, the contribution of microglial cells from other sources should also be considered. In addition to “true” or “*bona-fide*” microglia, which are of embryonic origin, the so-called “microglia-like cells” are hematopoietic cells of bone marrow origin that can engraft the mature brain mainly under pathological conditions. These cells implement great parts of the microglial immune phenotype, but they do not completely adopt the “true microglia” features. Because of their pronounced similarity, true microglia and microglia-like cells are usually considered together as one population. In this review, we discuss the origin and development of these two distinct cell types and their differences. We will also review the factors determining the appearance and presence of microglia-like cells, which can vary among species. This knowledge might contribute to the development of therapeutic strategies aiming at microglial cells for the treatment of diseases in which they are involved, for example neurodegenerative disorders like Alzheimer’s and Parkinson’s diseases.

## Introduction

Macrophages constitute a heterogeneous population of immune cells of the myeloid lineage, involved in phagocytic and other functions, like cell signaling and release of biologically active molecules. Specific tissue-resident macrophages are located in certain tissues/organs and represent particular macrophage subtypes.

**Figure 1:**
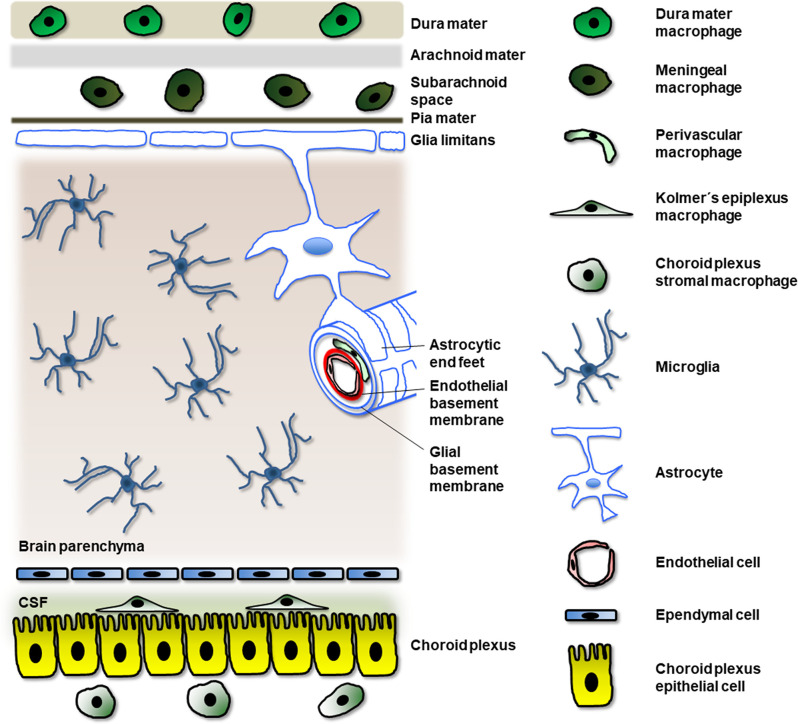
Localization of different myeloid cells related to macrophages in the CNS. Meningeal macrophages locate in the subarachnoidal space while other macrophages appear in other regions of the meninges (for example, dura mater macrophages). Perivascular macrophages localize between the endothelial and glial basement membranes (note that pericytes in the perivascular space of the blood vessel have not been represented for clarity). Kolmer’s epiplexus macrophages are found above the choroid plexus epithelial cells and in contact with the cerebrospinal fluid (CSF); they apparently derive from choroid plexus stromal macrophages located underneath the choroid plexus epithelial cells. Finally, the sole myeloid cells in the nervous parenchyma are microglial cells.

At least four types of macrophages have been identified in the mature and developing central nervous system (CNS; Figure 1): meningeal macrophages, perivascular macrophages, choroid plexus macrophages, and microglia (Goldmann et al., 2016; Herz et al., 2017; Lopez-Atalaya et al., 2018). Because the first three types are located at the borders of the nervous parenchyma, they are known as border-associated macrophages (BAM) or CNS-associated macrophages (CAM). In contrast, microglia are situated within the nervous parenchyma. Although BAM and microglia share some features, like the expression of the fractalkine receptor CX3CR1, both of them exhibit their own characteristics. For instance, microglia express the specific marker Tmem119, which is not expressed in BAMs (Li and Barres, 2018). A recent study reveals that both BAM and microglial cells derive from the yolk sac (YS) and acquire their phenotype depending on their genotype and location cues (Utz et al., 2020).

Hence, microglia are the resident macrophages of the nervous parenchyma. They can be distinguished from other tissue-resident and peripheral macrophages because of their embryonic origin from the YS, maintenance, and immunophenotype, as will be discussed in the next sections. They participate in the surveillance, development, homeostasis, protection and functional integrity of the CNS (Hanisch, 2013; Casano and Peri, 2015) being involved in many CNS disorders (Amici et al., 2017).

Microglial cells show a variety of morphological and immunological phenotypes in the developing CNS and mature healthy brain, with additional phenotypes in the pathological/aging brain (Deczkowska et al., 2018; Savage et al., 2019; Stratoulias et al., 2019). During development predominate microglial cells with a round morphology and short and broad processes (“amoeboid microglia”), while microglia in the adult brain frequently show thin processes with more or less profuse ramifications (“ramified microglia”; Cuadros and Navascues, 1998; Smolders et al., 2019). Cell marking experiments (e.g., Leong and Ling, 1992) have established that amoeboid microglia can transform into ramified microglia.

Techniques such as microarrays, bulk RNA-seq or single-cell RNA-seq, have revealed the presence of different subsets of microglia at the molecular level (Hammond et al., 2019; Gerrits et al., 2020; Masuda et al., 2020). They go through a stepwise developmental program during their maturation in which each phase is characterized by a different set of gene expressions (Matcovitch-Natan et al., 2016). The stages of microglial development in the mouse brain are: early microglia (approximately from E10.5 to E14), pre-microglia (approximately from E14 to the 1st postnatal week), and adult microglia (four or more postnatal weeks; Matcovitch-Natan et al., 2016; Thion et al., 2018b; Smolders et al., 2019). Apparently, miRNAs are involved in regulating the gene expression in microglia (Varol et al., 2017; Guo et al., 2019).

In addition to microglia of embryonic origin, the adult CNS also contains hematopoietic cells that can permanently engraft in the nervous parenchyma under certain conditions and acquire features similar to embryonic microglia (Varvel et al., 2012; Sevenich, 2018), as will be discussed in detail later in this review. The reported heterogeneity shown by microglial cells (Zia et al., 2020) may “hide” the presence of such cells that presumably were frequently included within the general term of “microglia”. Although microglial cells of embryonic origin share many markers, like CD11b, F4/80, CX3CR1, and Iba-1, with other macrophages and with the hematopoietic cells engrafting the nervous parenchyma in adulthood, the engrafting cells express high levels of CD45, whereas embryonic microglia express lower levels (Amici et al., 2017).

Gene expression analysis (reviewed in Gerrits et al., 2020) has revealed that the engrafted cells present molecular and functional differences compared with adult microglia of embryonic origin, despite the fact that they adopt a similar morphology (Bruttger et al., 2015; Bennett et al., 2018; Cronk et al., 2018; Shemer et al., 2018; Tables s 1 and 2). The term “microglia” (or “true microglia” or “*bona fide* microglia”) is reserved for myeloid cells from the YS that colonize the nervous tissue during embryonic stages and self-renew by proliferation, without the participation of new myeloid cells under physiological conditions. In contrast, myeloid cells derived from bone marrow (BM) that colonize the nervous parenchyma in response to pathological situations and establish a permanent population within the brain parenchyma (Cronk et al., 2018; Lund et al., 2018) are called bone marrow-derived microglia or microglia-like cells (MLC). In this article, we will use the term MLC, as in our opinion this reflects not only their different origin but also their slightly different characteristics.

**Table 1:** Representative genes with higher expression levels in YS-derived microglia (healthy adult) than in engrafted brain macrophages/microglia-like cells.

Gene	Protein class	Biological function	Function in microglia
P2ry12	Purinergic receptor	Cell migration Response to ADP/ATP	Regulation of microglial migration
Sall1	Transcriptional regulator	Developmental processes	Regulation of microglial identity and state
Siglec-H	Sialic acid receptor	Cargo receptor Cell adhesion Endocytosis	Recognition of phagocytic targets
Tmem119	Transmembrane protein	Biomineralization Differentiation	Unknown

**Table 2:** Representative genes showing higher expression levels in engrafted brain macrophages/microglia-like cells than in YS-derived microglia (healthy adult).

Gene	Protein class	Biological function	Function in microglia
*Axl*	Tyrosine kinase receptor	Regulation of phagocytosis and macrophage polarization Apoptotic cell clearance	Regulation of phagocytosis
*Ccr2*	Chemokine receptor	Chemotaxis	Chemotaxis of myeloid cells
*Cxcr4*	Chemokine (vgr. SDF-1) receptor	Chemotaxis	Nearly absent
*Cybb*	Cytochrome	Regulation of ROS generation	ROS generation

*Data in Table 1 and 2 from Shemer et al. (2018); Buttgereit et al. (2016); and UniProt database (https://www.uniprot.org/uniprot/, accessed 09 October 2021)*.

In this review, we discuss the origin, differences, and similarities of microglia and MLC. Although we mainly center on the microglia of mice, we also refer to other species, including humans.

## Microglia Are Cells of Hematopoietic Lineage

The first extensive description of microglial cells (del Rıo-Hortega, 1919, translated and annotated in Sierra et al., 2016; del Rio-Hortega, 1932) already proposed that microglial cells likely originated outside the nervous parenchyma. More recent evidence has strengthened this idea. Although different sources for the origin of microglial cells have been proposed (see Kaur et al., 2017), currently it is firmly established that microglial cells are related to the hematopoietic lineage.

Microglial cells are labeled with many reagents, which also mark hematopoietic cells (Table 3). The existence of reagents recognizing mature microglia but not embryonic microglia, e.g., anti-P2ry12 and anti-Tmem119 (Bennett et al., 2016), indicate that the transcriptome of mature microglia is different from the one of developing microglia and other tissue macrophages (Varol et al., 2017; Li et al., 2019).

**Table 3:** Common microglial markers.

Marker	Labeling on microglia	Labeling of non-microglial cells	Reference
CD68 immunohistochemistry	Endosomes and lysosomes of activated microglia.	Blood monocytes and peripheral macrophages.	Santos et al. (2010)
F4/80 immunohistochemistry	Amoeboid and ramified microglia (original batch). Amoeboid microglia (most current batches).	Peripheral macrophages.	Perry et al. (1985), Santos et al. (2008)
Iba-1 immunohistochemistry	Ca^2+^-binding protein in microglia (cytoplasm and membrane). Staining of amoeboid and ramified microglia.	Peripheral macrophages.	Ito et al. (1998)
Lectin ISB4 from *Griffonia simplicifolia*	Plasma and Golgi membranes. Strong labeling of amoeboid and poorly ramified microglia. Weak staining of fully ramified microglia.	Endothelial and blood cells. Peripheral macrophages.	Kaur et al. (2001)
Nucleoside diphosphatase (NDP) histochemistry	Plasma membrane of amoeboid and ramified microglia.	Endothelial and blood cells. Intracellular staining of neurons.	Murabe and Sano (1982)
P2ry12 immunohistochemistry	Purinergic receptor in ramified microglia.	No labeling in peripheral macrophages.	Butovsky et al. (2014)
Thiamine pyrophosphatase (TPP) histochemistry	Plasma membrane of ramified microglia.	Endothelial and blood cells. Intracellular staining of neurons.	Murabe and Sano (1981)
Tmem119 immunocytochemistry	Transmembrane protein in ramified microglia.	No labeling in peripheral macrophages.	Bennett et al. (2016)

Additional proofs were obtained from PU.1-mutant mice. PU.1 is a transcription factor product of the *Sfpi1* gene, which is expressed in cells of hematopoietic lineage. Lack of PU.1 results in mice devoid of macrophages, eosinophils, B cells, and the impairment of the development of neutrophils and T lymphocytes. Interestingly, PU.1^−/−^ mice do not possess microglia, confirming their hematopoietic origin (McKercher et al., 1996; Kierdorf et al., 2013a).

Mutations affecting the expression of hematopoietic factors also disturb microglial cells. Accordingly, macrophage maturation is impaired and microglia numbers are reduced in the osteopetrotic (op/op) mice, bearing a spontaneous null mutation in the gene that codifies the colony-stimulating factor-1 (CSF-1; Kondo et al., 2007). In contrast, *Csf1r*^−/−^ mice, that lack the CSF-1 receptor, show a nearly total depletion of microglia. This indicates that CSFR1 can also bind to another ligand important for microglia development, identified as interleukin-34 (IL-34; Greter et al., 2012; Wang et al., 2012). In fact, deletion of IL-34 resulted in the absence of microglia in a great part of the brain (Wang et al., 2012). Interestingly, not all microglial cells have the same requirements for CSF-1 and IL-34, as these two ligands affect differently microglia in the white and gray matter of the brain (Easley-Neal et al., 2019) and the development of mouse cerebellar microglia is not affected by IL-34 deficiency (Kana et al., 2019).

Therefore, multiple evidences support that microglia have a hematopoietic origin. In agreement with the conceptual framework of the mononuclear phagocyte system (van Furth et al., 1972), microglia are likely to derive from circulating monocytes (see Ginhoux and Jung, 2014). In fact, carbon labeling of blood monocytes results in the finding of some labeled amoeboid and ramified microglial cells in the corpus callosum of postnatal rats (Ling, 1979; Ling et al., 1980). Therefore, it was postulated that microglial cells, like other tissue-resident macrophages, were of monocytic origin (reviewed in Ginhoux et al., 2013).

However, this view has been challenged by reports using lineage-tracing techniques revealing that microglia, although of hematopoietic origin, originate independently of monocytes (reviewed, among many others, by Prinz et al., 2017 and Thion et al., 2018a), and that they are not replaced by BM-derived monocytes under physiological conditions, unlike other tissue-resident macrophages (Marquez-Ropero et al., 2020).

### Brief Description of Hematopoiesis in Mice

Because of the relation of microglial cells with hematopoiesis, we will shortly describe the development of hematopoiesis in the mouse, mainly focusing on macrophages.

Three different waves (or phases) of hematopoiesis have been described during mouse development. The first one, called the primitive hematopoietic program by Hoeffel and Ginhoux (2018), occurs in the YS and starts at E7–7.5. This phase is produced by hematopoietic precursors in the YS (denominated primitive myeloid precursors by McGrath et al., 2015) that differentiate as primitive erythrocytes, megakaryocytes, and macrophages.

The second wave begins around E8 and is produced by erythromyeloid progenitors (EMP, McGrath et al., 2015) located in the YS, giving rise to all types of hematopoietic cells (definitive erythrocytes and leukocytes), including fetal monocytes. Some EMP invade the liver rudiment and other hematopoietic organs, such as the spleen and thymus, during the development of the embryo and become the founder cells of the “transient definitive hematopoiesis” (Hoeffel and Ginhoux, 2018).

Finally, hematopoietic stem cells (HSC) produced in the aorta-gonad-mesonephros region (AGM) of the embryo start the third wave, or “definitive hematopoiesis”, at E10.5 (Hoeffel and Ginhoux, 2018). HSC will settle in the fetal liver (the main hematopoietic organ during most of the gestation period) and other hematopoietic organs of the embryo where they mature and differentiate to give rise to definitive erythrocytes and all myeloid cells, including monocytes. HSC from the fetal liver will finally colonize the BM, the only hematopoietic organ of the adult, where hematopoiesis starts at around the second postnatal week.

Therefore, macrophages are produced directly from their progenitors during the first hematopoietic and perhaps during the second wave, without passing through a monocyte stage, while they originate from monocytes during the second and third waves. Consequently, there are two different ways of macrophage production, in agreement with studies by Takahashi et al. (1989), (1996), which reveal the presence of macrophages in rodent embryos before the appearance of monocytes.

### Brief Description of Hematopoiesis in Non-mammalian Vertebrates

Although this scheme of hematopoiesis seems to be valid for other mammals, including humans (Tavian et al., 2010; Bian et al., 2020), differences affecting the development of macrophages and microglial cells have been reported in non-mammalian vertebrates. For example, the primitive hematopoiesis of the zebrafish originates in two different regions of the lateral plate mesoderm, anterior (rostral blood islands, RBIs) and posterior (posterior lateral mesoderm, PLM) to the yolk ball (equivalent to the YS of amniotes, as in anamniote vertebrates there is no YS of extra-embryonic tissues). The RBIs give rise essentially to macrophages, while the PLM originates fundamentally primitive erythrocytes. Pre-macrophages from the RBIs enter the yolk ball, where they differentiate, and later colonize the cephalic tissues, including the brain and retina (Herbomel et al., 1999; Bertrand et al., 2007; Bertrand and Traver, 2009; Gore et al., 2018). Therefore, the RBIs would be the site of origin of primitive macrophages and embryonic microglia (Herbomel et al., 2001; Gore et al., 2018). The second wave of hematopoiesis, apparently equivalent to the transient hematopoiesis described in the mouse, is produced by EMP arising in the posterior blood island (PBI). Finally, the definitive wave of hematopoiesis originates from the ventral wall of the dorsal aorta (VDA), producing HSCs capable of giving rise to all mature blood cells and microglial cells (Xu et al., 2015).

Birds are amniote vertebrates with development of hematopoiesis comparable to the one described in mice. In birds, YS macrophages and microglial cells are replaced during development by cells derived from intraembryonic precursors (Garceau et al., 2015).

## Origin, Development, and Maintenance of Microglia

The presence of brain macrophages and/or early microglia in the developing brain has been described in all vertebrates: zebrafish (Herbomel et al., 2001), amphibians (Goodbrand and Gaze, 1991), birds (Cuadros et al., 1993), rats (Sorokin et al., 1992), mice (Ginhoux et al., 2010; Ginhoux and Prinz, 2015) and humans (Andjelkovic et al., 1998; Verney et al., 2010). This widespread presence suggests that they are important for the development of the normal organization of the nervous system, sculpting the early stages of the nervous system (Ginhoux and Prinz, 2015). For example, the ablation of brain macrophage/microglia modifies the populations of sensory neurons (Angelim et al., 2018).

However, it has been reported that the lack of early brain macrophages and embryonic microglia does not produce gross abnormalities in the brain during embryonic stages (Patkar et al., 2021). Thus, embryos pharmacologically depleted of microglia survive until postnatal age without apparent morphological changes in the adult brain (Rosin et al., 2018). The authors detected an increase in neural cell death and cell debris in the developing brain of microglia-depleted embryos. However, this increase is no longer apparent in the adult brain, indicating that dead cells and cell debris are finally removed, suggesting that other cells replace the clearing role of microglia (Kierdorf and Prinz, 2017). In fact, despite the nearly complete depletion of microglia in *Csfr-1^−/−^* mice, the brain shows a normal organization during the prenatal period, although its cytoarchitecture is severely perturbed in the 3rd postnatal week (Erblich et al., 2011). Similarly, neurogenesis and overall patterning of the brain are not disturbed in embryos depleted of microglia during most parts of embryonic development (Squarzoni et al., 2014). The authors indicate, however, that some cortex interneurons and wiring processes, such as the dopaminergic projection invading the striatum, are affected by the absence of microglia. Other authors show that the absence of microglia also disturbs the fasciculation of callosal axons (Pont-Lezica et al., 2014).

Thus, the presence of brain macrophages and/or microglial cells seems to be dispensable for the gross development of the brain and the lack of microglia does not produce evident abnormalities in the brain architecture during the embryonic period.

### Microglial Origin From the Yolk Sac

Although previous studies have already proposed that microglial cells might be of YS origin (Cuadros et al., 1993; Alliot et al., 1999), direct evidence of this origin was reported in 2010 using genetic labeling of microglial precursors (Ginhoux et al., 2010) using a transgenic *Runx1*^Mer-Cre-Mer^mice Runx1 is a transcription factor needed for hematopoiesis (Yzaguirre et al., 2017; Menegatti et al., 2019) whose expression at E7.5 is restricted to the YS; one day later, *Runx1* expression is observed in the AGM region (Samokhvalov et al., 2007). Administration of 4-hydroxytamoxifen (4’OHT) to induce recombination between E7.0 and E7.5 produces permanent labeling of more than 30% of the microglial cells in the adult brain; in contrast, circulating leukocytes and monocytes are not labeled. The injection of 4’OHT after E7.5 results in a progressive reduction in the number of labeled microglial cells in the adult and the labeling of HSC-derived cells (Ginhoux et al., 2010). Thus, the authors conclude that microglial cells originate from cells produced around E7.5 in the YS and not from blood monocytes, i.e., that microglial cells derive from primitive myeloid precursors, corresponding to the first wave of hematopoiesis.

Other lineage tracing studies using similar approaches obtain slightly different results, as the mapping is related to the particular expression pattern of each gene: *Runx1* expression is limited to precursors budding from the hemogenic endothelium, while *c-Kit*, another gene used in the mapping studies (see below), is expressed by all hematopoietic progenitors (Hoeffel and Ginhoux, 2015). The analysis of Kit^Mer-Cre-Mer^ mice shows that similar numbers (≈50%) of microglial cells are labeled after injection at E7.5 or E8.5 (Sheng et al., 2015), indicating that microglia could derive from primitive myeloid precursors and EMP. Gomez Perdiguero et al. (2015) discriminate between cells originated from YS-derived cells and HSC progenitors using *Csfr1*^Mer-Cre-Mer^ and *Flt3*^Cre^ mice (*Flt3* is mainly expressed in cells derived from HSC progenitors). Confirming former studies (Schulz et al., 2012), this report reveals that intraembryonic HSC account only for a minor fraction of microglia. Similarly, another study concludes that most macrophages, except microglia, initially derive from embryonic precursors that are later replaced by BM-derived macrophages (Sheng et al., 2015).

The above reports used genetic lineage tracing studies, a very powerful tool to investigate the fate of progenitors *in vivo*, but with certain limitations (McGrath et al., 2015). For instance, the efficiency of recombination of developing cells will produce distortions on the contribution of progenitors to the final population (Samokhvalov et al., 2007; Gomez Perdiguero et al., 2015), explaining why a fraction of macrophages remain unlabeled. In this sense, it is worthy to note that the authors of the first report (Ginhoux et al., 2010) affirm that their model probably underestimates the contribution of *Runx1+* cells to adult microglia and that they cannot exclude the contribution of non-labeled precursors to the ontogeny of microglia. Moreover, a significant proportion (7–10%) of adult HSC were of extra-embryonic origin, raising the question that part of the *Runx1+* labeled microglia in the adult are likely to originate from YS precursors that participate in the definitive hematopoiesis (Samokhvalov et al., 2007). In this sense, a review about the ontogeny of resident macrophages (Ginhoux and Guilliams, 2016) states that, “it remains possible that a minor population of adult microglia, […] might derive from non-YS progenitors recruited later during development”. Additional caveats about the results obtained from lineage studies have been expressed (Epelman et al., 2014; McKinsey et al., 2020).

Other experiments establish that the first macrophage population appearing during development are of YS origin and evolve independently of the transcription factor Myb, a critical regulator of hematopoiesis; the second population is constituted by macrophages derived from HSCs and require Myb. Microglial cells originate from the first population and are not replaced by Myb-dependent hematopoietic cells, as normal numbers of microglial cells are detected in *c-Myb*^−/−^ mice (Schulz et al., 2012).

The first microglia precursors are EMP detected in the YS at E8 (CD45^−^, c-kit^+^). Later on, A1 cells (CD45^+^, c-kit^−^, CX3CR1^−^) emerge, which give rise to microglia precursor A2 cells (CD45^+^, c-kit^−^, CX3CR1^+^). Both A1 and A2 cells appear in the YS and are able to differentiate into microglial cells in organotypic hippocampal slice cultures. A2 cells enter the cephalic mesenchyme and produce microglial cells in the developing brain from E9.5 and onwards. The absence of PU.1 affects both A1 and A2 cells, resulting in the complete lack of microglial cells. In contrast, the lack of *Irf8* gene, encoding another transcription factor of myeloid cells, only reduces the number of microglia. Careful analysis shows that A1 cells are unaffected by the lack of Irf8, but that A2 cells are severely depleted under the same conditions, suggesting a role of Irf8 in microglial maturation (Kierdorf et al., 2013a). The remaining A2 cells can proliferate and produce microglial cells, however with phenotypical and morphological alterations (Minten et al., 2012).

Other studies reinforce the idea that microglial cells and embryonic macrophages are related. For example, E10.5 *Runx1^−/−^* embryos, which do not contain embryonic macrophages, also lack microglia precursors (Ferrero et al., 2018) and the lack of embryonic macrophages accompany the depletion of microglial cells in the developing brain (Angelim et al., 2018; Rojo et al., 2019).

### Entry and Colonization of the Developing Brain

**Figure 2:**
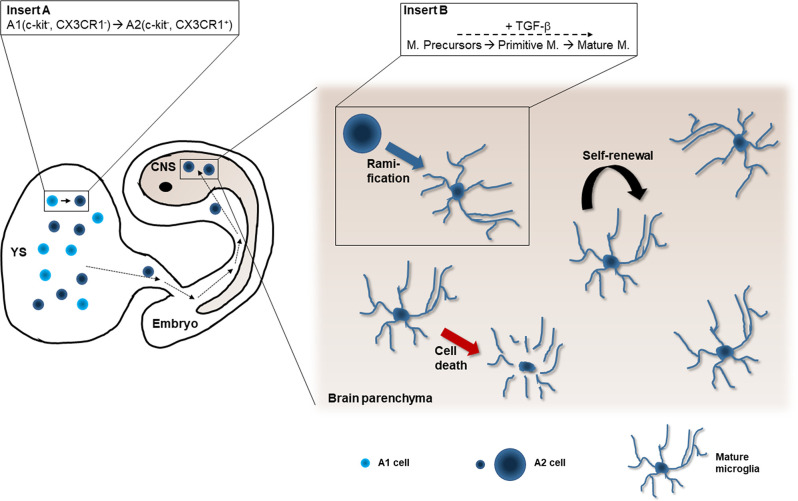
Microglial origin from the yolk sac. Microglia precursors are produced in the yolk sac (YS; A1 and A2 cells, Kierdorf et al., 2013a; insert **A**). A2 cells enter the developing embryo and colonize the cephalic mesenchyme (dashed black arrows). Some of them pass into the central nervous system (CNS) anlagen where they differentiate into microglia (blue arrow). The differentiation process is progressive (insert **B**): Microglial precursors (also called early microglia in Matcovitch-Natan et al., 2016) transform into primitive microglia (called pre-microglia in Matcovitch-Natan et al., 2016) and finally acquire the ramified phenotype of mature microglia (adult microglia in Matcovitch-Natan et al., 2016). This process depends on the cytokine TGF-β (Butovsky et al., 2014; Utz et al., 2020). The microglial population maintains stable in the healthy brain by means of proliferation (curved black arrow) and programmed cell death (red arrow; Askew et al., 2017).

Microglia progenitors from YS invade the developing CNS to become microglial cells at E9.5 (Ginhoux et al., 2010; Kierdorf et al., 2013a; Figure 2), before the establishment of the blood-brain barrier (BBB), which begins around E15 in mice (Daneman et al., 2010). As the developing brain is colonized by YS macrophages before any other structures of the embryo (Sorokin et al., 1992), it is reasonable to assume that these cells are attracted to the developing brain before they are attracted to any other tissue. The invasion of the developing brain raises questions about whether there is a specific subpopulation of embryonic macrophages for invading the neuroepithelium and about the identity of factors eliciting the entry within the developing CNS.

Regarding the first question, a subtype of embryonic macrophages, specific for entering the brain and giving rise to microglia, is described in the zebrafish (Rossi et al., 2015). However, the existence in mammals of a subpopulation of macrophages in the embryo devoted to colonizing the brain remains to be demonstrated.

Blood circulation can also affect macrophage colonization, as shown by the fact that *Ncx-1^−/−^* mice, characterized by loss of heart beating and impaired development of the circulatory system, have no detectable brain macrophages/microglia at E9.5–10.5 (Ginhoux et al., 2010). In contrast, early colonization of zebrafish midbrain by microglia precursors is described as circulation-independent (Xu et al., 2016). In addition, entry into the avian CNS seems to be independent of the establishment of circulation, as macrophage invasion precedes the presence of vascular buds into the neuroepithelium (Cuadros et al., 1993) and cells of YS origin colonize the avian avascular retina (Cuadros et al., 1991). Studies on the early appearance of brain macrophages in rodents and humans (e.g., Sorokin et al., 1992; Andjelkovic et al., 1998; Kierdorf et al., 2013a; Menassa and Gomez-Nicola, 2018) do not show a clear correlation between the appearance of these cells within the neuroepithelium and blood vessel development. It is possible that the emigration of early macrophages from the YS into the embryo would require functional circulation (Stremmel et al., 2018). However, these cells might leave the blood vessels after reaching the embryo mesenchyme and migrate to the neuroepithelium independently of the vasculature.

Cell death has frequently been associated with the presence of macrophages. Lysophosphatidylcholine, a lipid product released by apoptotic cells that attracts monocytic cells (Lauber et al., 2003), promotes the migration of macrophages into the zebrafish midbrain (Xu et al., 2016). Other authors have also reported that altering the rate of cell death also changes the number of macrophage/microglial cells in the zebrafish brain (Casano et al., 2016; Wu et al., 2018). It has been proposed that microglia precursors colonize the zebrafish brain by two types of mechanisms: one apoptosis-dependent and another one apoptosis-independent. The first one would happen early in development and might be driven by chemoattractants released by dying cells produced during early neurogenesis. The apoptosis-independent migration might occur later during development and depend on other factors, such as IL-34 (Xu et al., 2016; Wu et al., 2018). In higher vertebrates (including rodents and mammals), brain macrophages appear independently of the abundance of previous cell death (Cuadros et al., 1993; Santos et al., 2008; Toyoshima et al., 2012), although some reports affirm that microglial cells appear coincidentally with cell death in the rat (Ashwell, 1991) and human (Rakic and Zecevic, 2000) telencephalon.

The spreading of microglial cells through the nervous parenchyma may be influenced by the distribution of migratory substrates, such as nerve fibers, blood vessels, or neuroglia cells (Navascues et al., 1995; Marin-Teva et al., 1999; Sanchez-Lopez et al., 2004; Mondo et al., 2020), and by chemoattractive and chemorepellent molecules (Smolders et al., 2019). For example, the entry of microglial cells into the developing quail retina is related to extracellular ATP/ADP or UTP/UDP (Martin-Estebane et al., 2017). In this sense, intracellular Ca^2+^ waves, which are elicited by ATP and propagate from cell to cell, affect the migratory response of microglial cells (Sieger et al., 2012; Morales-Ropero et al., 2021), and might be involved in the colonization of the developing nervous tissue.

The microglial spreading finally results in their “tiled” distribution in the adult brain, in which each cell and their processes occupy their own spatial domain, not overlapping with other microglia. One of the proposed mechanisms contributing to the acquisition of this distribution is contact repulsion between individual microglia (Hammond et al., 2021).

### Heterogeneity of Microglia

Microglia is not a homogeneous population (Doorn et al., 2015; Kana et al., 2019; Tan et al., 2020). While all microglial cells express core “microglial” genes, as *Cx3cr1*, *P2ry12*, or *Tmem119*, microglia from specific brain regions express other genes at different levels, e.g., cerebellar microglia show an increased expression of genes related to phagocytosis and clearance when compared to cortical microglia (Ayata et al., 2018).

The features of different populations of microglial cells can appear from an initially homogeneous population that develops into different cells depending on environmental cues. Alternatively, microglial precursors can be a heterogeneous population comprising different subtypes, each one with intrinsic features, that gives rise to particular microglial cells (Stratoulias et al., 2019). In the second case, the existence of different microglial populations could partially be due to the different origin of dissimilar cells (Tan et al., 2020). In line with this, at least one study determines that a subset of microglial cells has a particular origin in mice (De et al., 2018). The authors reveal the existence of two different subpopulations of microglia: a larger population of “canonical” microglia that did not express *Hoxb8*, and *Hoxb8*-expressing microglia (about 25% of adult microglia) that derive from YS precursors, seed the AGM and fetal liver, and enter the developing brain at E12.5, significantly later than non-*Hoxb8* microglia (De et al., 2018).

### Maintenance of the Microglial Population

Once within the neuroepithelium, microglial precursors are influenced by factors relevant for the maturation and/or maintenance of the microglia population, as members of the transforming growth factor-β (TGF-β) family (Butovsky et al., 2014; Bohlen et al., 2017), and acquire a “microglia signature”, that include the expression of genes like *Sall1*, *P2ry12* and* Tmem112* (Amici et al., 2017; Butovsky and Weiner, 2018; Bohlen et al., 2019).

To maintain the YS lineage in microglia as a self-autonomous population it would be required that they could maintain themselves without appreciable renewal from HSC. This can be achieved either by a proliferation of microglia, continuously replacing cells with ones of their own population, or by microglia being a long-lived population with a low proliferation rate. Initially, it was postulated that microglial cells were long-lived cells that could persist the entire lifespan of mice or humans (Fuger et al., 2017). However, a detailed study has revealed that their proliferation rate is higher than previously thought (Askew et al., 2017): around 0.69% of the microglia population is embarked in proliferation in mice at each moment, implying that the entire microglial population is replaced in about 96 days (the estimated value in humans is around 2%). Therefore, individual microglial cells frequently replicate, although the microglial population as a whole may be considered as long-living. Receptors like CSFR1 and IL-1R regulate the replication of microglia (Bruttger et al., 2015; Askew et al., 2017). The steady density of microglial cells is maintained during adult life by coordinating proliferation and cell death (Askew et al., 2017).

### Alternative Origins of Microglia in the Developing Brain

Despite the results reported above, other data raise concern about the hypothesis that the YS would be the exclusive site of origin of microglia.

In Vav1-Cre^+^:dicer knock-out mice the intraembryonic hematopoiesis is impaired, while YS hematopoiesis is not affected (Fehrenbach et al., 2018). Although similar numbers of microglial cells are reported during the embryonic development in WT and KO animals, about 40% less myeloid cells are counted in KO mice at P1, suggesting that a part of the microglia population present in the brain at this age does not derive from YS. Presumably, all or at least part of the myeloid cells of non-YS origin observed at P1 in this report correspond to the perinatal wave of infiltrating monocytes that do not contribute to the microglia population at adulthood (Askew et al., 2017).

Other reports also indicate that microglial cells may also derive from non-YS cells. The injection in E6.5-E7.5 mice with a blocking antibody against CSFR1 depletes YS macrophages but leaves the population of circulating monocytes unaltered. This procedure results in the total lack of microglial cells until E14.5, but microglia partially repopulate the brain by E17.5 and similar microglia numbers are found in adult brains of control and treated mice (Squarzoni et al., 2014; Hoeffel et al., 2015). Therefore, the depletion of microglial cells in the developing CNS is compensated at the end of development by the progressive appearance of new microglial cells that perhaps are related to fetal monocytes and that adopt microglial characteristics in the brain parenchyma (Ginhoux and Guilliams, 2016; Chen et al., 2020). This fact might indicate a non-YS source of microglia.

As mentioned before, the CNS of PU.1^−/−^ mice are devoid of microglia. However, the microglial population in the spinal cord of PU.1^−/−^ mice can be reconstituted after transplantation of BM cells from WT mice into neonatal PU.1^−/−^ pups. This indicates that non-YS cells can invade the CNS and acquire the characteristics of microglial cells (Beers et al., 2006). Another work (Bennett et al., 2018) reveals that intraperitoneal injection of BM cells from WT mice into P1 *Csfr1^−/−^* mice, which lack microglia, results in the appearance of engrafted Tmem119+ cells in the brain of the host without microglia.

In birds, most adult microglia apparently do not derive from YS precursors. When YS-derived fluorescence-labeled cells are injected into the circulation of chick embryos before the establishment of intraembryonic hematopoiesis, many ramified microglial cells of YS origin appear until hatching. However, only occasionally labeled microglia are observed in the brain of newly hatched birds and no fluorescence is observed in the brains of adult birds. On the contrary, abundantly labeled microglia are seen in adult brains when labeled BM cells from newly hatched birds were injected before the start of intraembryonic hematopoiesis (Garceau et al., 2015). These results suggest that in the chick brain macrophages/microglial cells are first produced from cells of YS origin, but that they are later replaced by cells of BM lineage.

Zebrafish microglial cells may have alternative origins. Genetic labeling of restricted areas at different time points revealed that microglia of embryonic/larval stages originated from the RBI (see “Brief Description of Hematopoiesis in Non-mammalian Vertebrates” section). In contrast, most adult microglia are produced from the VDA during the definitive hematopoiesis (Xu et al., 2015). Microglia produced during the definitive hematopoiesis, in contrast to embryonic microglia, depend on the expression of the transcription factor c-Myb (Ferrero et al., 2018).

Differences between the origin of microglia in the mouse and zebrafish may be due to interspecific differences, but also open the question if in mammals alternative sources of microglia exist that still have not been identified (Thion and Garel, 2017). In any case, the ontogeny of brain macrophages/microglia is likely to be more complex than currently assumed (Prinz et al., 2019) and could encompass several sources during development.

To summarize, the current consensus is that microglial cells originate from a cell lineage appearing in the YS from primitive myeloid precursors or EMP. Several studies, however, indicate that some microglia, in mice and other vertebrates, might have an alternative origin during development, without contradicting that a great part of the microglial population originates in the YS.

## Microglia-Like Cells (MLC)

Myeloid cells enter the nervous parenchyma during the whole life span of an organism and some of them apparently transform into cells resembling microglia, the MLC. Despite their resemblance to microglia, they do not become true microglial cells, because they differ in origin, morphological and functional aspects.

Engraftment of myeloid cells in the nervous parenchyma depends on the capacity of blood-derived cells to enter into the CNS. The BBB limits and hampers the entry of blood cells and most soluble blood components into the nervous parenchyma (Wettschureck et al., 2019). Therefore, the BBB precludes a continuous replacement of microglial cells of embryonic origin by cells arising from the BM. Similarly, the MLC population is also maintained by local proliferation (Askew et al., 2017). The consequence is that both microglia and MLC establish a common “macrophage network” within the nervous parenchyma.

### Engraftment of Myeloid Cells in Healthy CNS

Although several studies (Ajami et al., 2007, 2011) discard the hypothesis that BM-derived monocytes might be a source of microglia in the adult healthy brain, many others show occasional appearance of MLC related to circulating myeloid cells. For example, labeled amoeboid cells are detected in the corpus callosum of postnatal rats after intracellular marking of blood monocytes with colloidal carbon and they occasionally adopt a ramified, microglia-like morphology (Kaur et al., 2001).

The question if circulating cells could engraft in the CNS, was addressed using chimeric mice in which host myeloid cells were depleted by irradiation (usually 9–11 Gy) before transplantation of donor BM or blood circulating labeled cells (Soulet and Rivest, 2008). It should be noted that host microglial cells are resistant to radiation and, therefore, are not depleted. Various studies have revealed that cells of donor origin engraft in the nervous parenchyma several days after the transplant (e.g., Kennedy and Abkowitz, 1998), some of them acquiring a microglial morphology (Priller et al., 2001; Simard and Rivest, 2004; Xu et al., 2020). However, if the head of the recipient mouse is protected during irradiation, the brain shows no engraftment, while the unprotected spinal cord is infiltrated by numerous myeloid cells (Mildner et al., 2007). This raises the concern that the engraftment of cells observed after irradiation would not be a physiological event, because this procedure artificially enhances the engraftment of cells by perturbing the BBB and producing neuroinflammation. Moreover, the BM transplant probably releases HSC into the circulating blood, which in normal conditions would be restricted to the BM, and, therefore, would not be able to originate MLC (Larochelle et al., 2016).

In the parabiosis model (Ajami et al., 2007; Liu et al., 2007; Hashimoto et al., 2013) the blood circulation of two mice are surgically joined, so that the fate of the circulating cells of one of the two parabionts, for example expressing the green fluorescent protein (GFP), could be determined in the other GFP-negative mouse. This method avoids any alteration of the BBB and the artificial release of BM-residing HSC into the blood. However, as no myeloablation is carried out, precursors from each partner are supposed to compete with similar cells from the other animal, possibly resulting in an underestimation of the rate of engraftment. Under these conditions no (Ajami et al., 2007) or very low (Massengale et al., 2005) engraftment in the healthy brain is reported. In a modification (Ajami et al., 2011), one of the partners (the GFP-negative mouse) is irradiated to achieve myelodepletion, whereas the other one is protected. Although nearly 80% of GFP-positive circulating cells are found in the peripheral blood of the GFP-negative mouse, there is no noticeable colonization of the irradiated CNS by GFP-positive cells. This suggests that the engrafting cells in the traditional irradiation-BM transplantation model are HSC that are not released into the blood circulation in parabiosis.

Chemotherapeutic drugs such as busulfan have been used to deplete host myeloid cells avoiding the side effects of irradiation (Larochelle et al., 2016; Youshani et al., 2019). Even though the busulfan treatment results in high levels of chimerism in the peripheral blood, conflicting results about the efficiency of microglial engraftment after this treatment are reported. One study reports that far less MLC appear after busulfan treatment than in whole body irradiated chimeric mice (Kierdorf et al., 2013b). According to this work, busulfan induces less alteration of the BBB and less inflammation in the brain than irradiation. However, another report (Capotondo et al., 2012) indicates that more MLC of donor origin appear after busulfan immunodepletion than after irradiation. Similar regimens of irradiation (11 Gy) are used in both articles, but the busulfan treatments are different (3 × 30 mg/kg in Kierdorf et al., 2013b) and 4 × 25 mg/kg in Capotondo et al., 2012). It has been reported that busulfan at concentrations around 100 mg/kg produces an artificial engraftment of myeloid cells (Larochelle et al., 2016; Youshani et al., 2019), an amount reached by Capotondo et al. (2012) but not by Kierdorf et al. (2013b). This could in part explain the described differences. Interestingly, Capotondo et al. (2012) also report that treatment with a busulfan derivative (Triosulfan) unable to cross the BBB does not result in any appreciable engraftment of myeloid cells, suggesting that the engraftment into the brain tissue requires an effect of the myeloablative drug on the nervous parenchyma.

**Figure 3:**
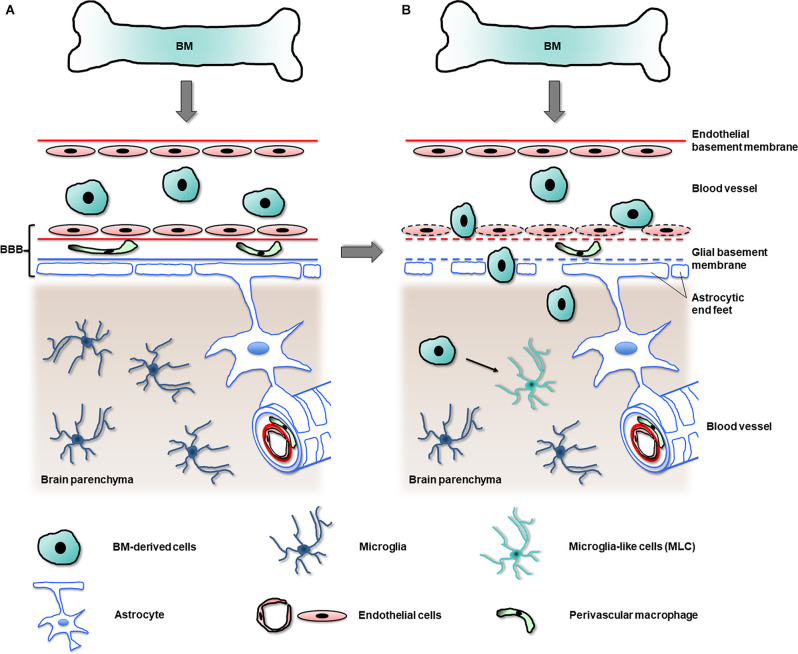
Migration of BM-derived cells into the CNS in health and disease. In the healthy brain **(A)** an unimpaired blood-brain barrier (BBB) hampers the passage of most circulating cells originating in the bone marrow (BM) into the brain parenchyma, avoiding the replacement of the original microglia. In the diseased brain **(B)**, in contrast, the BBB becomes leaky (as indicated by dashed lines) and allows the entry of BM-derived cells into the brain parenchyma. In regions with partial or total depletion of microglia, some of the BM-derived cells become microglia-like cells (MLC, arrow), which adopt many features of original microglia.

In summary, although the experimental procedures used to determine the entry of myeloid cells into the adult healthy CNS are likely to affect the recruitment of BM-derived microglia, the data strongly suggest that the mature microglial population is mostly permanent and autonomously self-renews with scarce contribution from peripheral BM-derived cells in the healthy brain (Figure 3A).

### Engraftment of Myeloid Cells in the Pathological CNS

In contrast to physiological conditions, numerous cells of myeloid lineage appear in the nervous parenchyma in pathological situations such as in Alzheimer’s disease models, experimental autoimmune encephalitis (EAE, the mouse model of multiple sclerosis), or traumatic injury. This implies that the diseased or preconditioned brain is in general far more receptive than the healthy brain to the entry of myeloid cells into the nervous parenchyma.

Some reports claim that the myeloid cells that penetrate the nervous parenchyma do not become microglial cells, but only give rise to macrophages that disappear after the end of the pathological situation and that the microgliosis observed in pathologies as facial nerve axotomy and EAE would be the only consequence of the expansion of resident microglia (Ajami et al., 2007, 2011). However, many publications indicate that at least some of the engrafted cells adopt microglial features in the parenchyma (Priller et al., 2001; Soulet and Rivest, 2008; Kierdorf et al., 2013b; Bennett et al., 2018). It has been shown that the engrafted myeloid cells persistently colonize the parenchyma for more than 30 weeks after a lipopolysaccharide (LPS) insult and acquire radio-resistance as well as many other traits of microglial cells, although they failed to adopt a complete microglial identity (Shemer et al., 2018).

Two facts seem to be decisive for the entrance of myeloid cells into the nervous parenchyma: (i) the permeability of the BBB; and (ii) the previous presence of microglial cells. As later described, these facts are not independent but rather closely linked.

#### Blood-Brain Barrier Permeability

The BBB permeability is compromised in most pathological situations (Dinet et al., 2019; Thurgur and Pinteaux, 2019; Giannoni et al., 2020; Yu et al., 2020). This allows the passage of blood cells into the nervous parenchyma and is likely to be the reason why many engrafted cells appear in the diseased brain. Interestingly, the number of engrafted myeloid cells after busulfan treatment (with minimal alteration of the BBB) is similar in the lesioned and contralateral sides of mice with facial nerve axotomy. However, far more myeloid cells of donor origin appear in the lesioned side than in the unlesioned one in irradiated mice (with altered BBB; Kierdorf et al., 2013b), reinforcing the idea that BBB integrity plays a decisive role in determining the entry of blood-derived cells into the parenchyma.

However, BBB leakiness is not the sole factor contributing to the entry of myeloid cells into the nervous parenchyma (Ajami et al., 2007), and additional signals from the diseased nervous parenchyma can promote the invasion of myeloid cells:

Several cytokines/chemokines produced in the injured brain alter the permeability of the BBB, allowing the passage of substances and cells that are normally excluded from the brain parenchyma (Pan et al., 2011). Among the molecules that can be involved in the recruitment of BM cells into the nervous parenchyma are the chemokines CCL2 (MCP-1), CX3CL1, and CXCL10 and adhesion molecules such as LFA-1, ICAM-1, VCAM-1, and E-selectin (Larochelle et al., 2016; Li et al., 2019). One of the main chemoattractants of myeloid cells is CCL2, which promotes the adhesion of blood monocytes and macrophages to the endothelial wall and subsequent infiltration to the brain parenchyma. This chemokine is released by numerous cells, including astrocytes, microglial cells, and neurons (Banisadr et al., 2005; Deshmane et al., 2009), and has been implicated in neuropathological situations (Reaux-Le Goazigo et al., 2013). In photodamaged retinas, macrophages and activated microglial cells produce the pro-inflammatory cytokine IL-1β that in turn promotes the expression of CCL2 (and other chemokines) by Müller cells and retinal pigment cells, associated with the accumulation of monocytes-macrophages in the damaged retina (Natoli et al., 2017). CCR2, the CCL2 receptor, seems to determine the recruitment of myeloid cells into the nervous parenchyma, as mice lacking this receptor show substantially less recruitment of myeloid cells than WT mice (Mildner et al., 2007; Lund et al., 2018). Interestingly, the development of microglia in the embryo does not depend on the CCL2/CCR2 system, as *Ccr2^−/−^* mice do not show different numbers of microglia than WT mice (Mildner et al., 2007).

#### Microglia Depletion

It has been proposed that monocyte-derived macrophage precursors compete with embryonic-derived macrophages to occupy the niche corresponding to each macrophage type (Guilliams and Scott, 2017; Guilliams et al., 2020). According to this idea, the previous presence of microglial cells in the parenchyma would limit the engraftment of myeloid cells into the nervous parenchyma. In the normal brain, microglial cells occupy entirely the “microglial niche” apparently hampering the engraftment of MLC. This would explain the scarcity of engraftment from BM-derived cells after irradiation because the radio-resistant original microglia are always present. However, the engraftment could take place if all or part of this niche was void, as it occurs in pathological situations. This idea has been addressed by depleting the microglial population.

Genetic depletion of microglia (Green et al., 2020) can be achieved by using animals lacking essential genes for the development or maintenance of microglia, such as *Sfpi1* and *Csf1r*. A detailed study using *Csf1r*^−/−^ mice shows the nearly total absence of microglia during the development (Erblich et al., 2011). However, disparate results are obtained in adult mice depending on the brain region: while microglia depletion is nearly complete (99%) in the thalamus and hypothalamus, it reaches only 65% in the piriform cortex. It has to be taken into account that the expression of these genes is not specific to microglial cells and can affect numerous organs, making it difficult to distinguish between the direct effects of the lack of microglia and those due to indirect effects on microglia due to the alteration of other organs and the hematopoietic system (Waisman et al., 2015). Recently, it has been found that mice without *fms-*intronic regulatory element (FIRE) enhancer of Csf1r expression lack microglia and resident macrophages in several organs, such as skin and kidney, but have normal myeloid populations in the peripheral blood, representing a useful model of genetic depletion of microglia without disturbing blood leukocytes (Rojo et al., 2019).

To establish models to eliminate microglial cells *in vivo* with fewer effects on the entire organism, conditional and pharmacological methods have been developed. For the conditional depletion of microglia, the expression of a suicide gene [such as herpesvirus thymidine kinase (HVTK) or the diphtheria toxin receptor (DTR)] is limited to macrophage/microglial cells in transgenic mice. The death of the cells expressing the suicide gene is triggered by the administration of a toxin (ganciclovir or diphtheria toxin, respectively) at defined times. These methods can eliminate around 90% of microglial cells (Waisman et al., 2015).

Most pharmacological methods to deplete microglia rely on CSFR1 inhibitors because the ligands of this receptor are needed for the maintenance of microglial cells (Erblich et al., 2011; Keshvari et al., 2021). CSFR1 inhibitors PLX3397 or PLX5622 deplete up to 99% of the microglial cells (Elmore et al., 2014; Huang et al., 2018b; Green et al., 2020). Usually, it is assumed that this treatment does not affect the myeloid and lymphoid compartments of blood and BM. However, this idea has recently been questioned, showing that PLX5622 treatment caused long-term effects on hematopoietic cells in BM and on BM-derived macrophages (Lei et al., 2020). This observation must be considered when interpreting data from animals treated with CSFR1 inhibitors, as CNS engraftment of blood cells might be perturbed by the treatment.

The microglial population recovers around 7 days after the end of the treatment (Elmore et al., 2014; Bruttger et al., 2015; Huang et al., 2018b). What is the origin of the cells which repopulate the microglial compartment? Most authors support the idea that the microglial population recovers from cells resident in the CNS (Elmore et al., 2014; Huang et al., 2018b). In this sense, mice in which microglia are depleted with PLX5622 and irradiated with head covering (and therefore with a minor alteration of the BBB) have minimal engraftment of BM-derived cells and the brain is repopulated by endogenous microglia. On the contrary, when the head covering is omitted and the BBB is likely to be affected, there is a robust engraftment of BM-derived cells, which participate in the repopulation (Cronk et al., 2018). This suggests that repopulating microglia are of intrinsic origin, without large participation of cells of BM origin unless the BBB would be affected (Bruttger et al., 2015; Zhan et al., 2019).

Originally, it was proposed that the repopulating cells derived from the proliferation of microglia precursors in the brain and were characterized by the expression of nestin (Elmore et al., 2014). Other authors using genetically labeled cells affirm that new microglial cells result from the proliferation of the scarce microglial cells (around 1%) remaining after the depletion (Huang et al., 2018b). They also state that the expression of nestin is not linked to a subgroup of microglial cells, but rather transiently found in all proliferating microglia and in myeloid cells outside the CNS. Similar conclusions are drawn in a study using DTR mice (Bruttger et al., 2015), which moreover indicates the presence of clusters of proliferating microglia in the brain that disappear after reaching the normal density of microglia. A study combining depletion of microglia and labeling of proliferating cells (Zhan et al., 2019) reinforces the idea that nestin expression is a transient marker of immature microglia and not a marker of microglial precursors.

The retina is a suitable region to study the effect of local depletion of microglia. According to Huang et al. (Huang et al., 2018a), PLX5622 treatment eliminated all retinal microglia, impeding the repopulation from remaining microglial cells in the retina. In this case, the repopulating cells derive from the proliferation and migration of residual microglia in the optic nerve and from macrophages from the ciliary body or iris (cells of BM origin). Local depletion of microglia occurs in some pathologies of the retina. For instance, after light damage of the retina, resident microglia leave the inner regions of the retina and migrate towards the photoreceptor cell layer (Santos et al., 2010; O’Koren et al., 2019). The vacant space left by migrating cells is occupied by cells coming from other regions of the CNS or by monocyte-macrophages from the ciliary body or iris (Huang et al., 2018a; Zhang et al., 2018; McPherson et al., 2019). Interestingly, a study combining microglial depletion and retinal injury shows that monocytes entering the retina after injury are resistant to CSFR1 inhibition, while cells repopulating the retina in uninjured retinas remain sensitive to the same inhibitor (Paschalis et al., 2018). These results suggest that different progenitors of microglia or MLC occupy the microglial niche in healthy and pathological nervous tissue.

A recent report (Xu et al., 2020) indicates that more than 90% of the microglial population is replaced by MLC of BM origin when original microglia have been depleted before myeloablative irradiation and inoculation of BM cells. In line with this, Cronk et al. (2018) report that persistent partial depletion of microglia induces the engraftment of blood circulating myeloid cells into the nervous parenchyma. In another model that combines microglia depletion with irradiation of head-protected mice or busulfan treatment and BM transplant (Lund et al., 2018), the microglial population consists of a mixture of “*bona fide*” microglia and MLC derived from circulating leukocytes. Therefore, BM-derived cells may significantly participate in the recovery of the microglial population when the microglial niche is not occupied without the need for apparent disturbance of the BBB.

How can microglia depletion affect the colonization and engraftment of circulating cells? Beforehand, we must consider that the elimination of microglial cells is not a physiological event, but it is likely that it alters the normal CNS environment. Some authors defend that microglia depletion does not induce great changes in CNS cells or cytokine release (Parkhurst et al., 2013; Elmore et al., 2014), but others describe an increase of pro-inflammatory cytokines (Bruttger et al., 2015; Lund et al., 2018). As reported above, the release of cytokines/chemokines affects the entry and engraftment of BM-derived cells into the CNS, because of the chemotactic attraction of circulating cells and a BBB compromise. Therefore, the absence of microglia might induce increasing levels of cytokines/chemokines and other molecules, such as ROS, that in turn might condition the colonization of the CNS parenchyma by hematogenous cells (da Fonseca et al., 2014).

In addition, microglia depletion can directly affect the integrity of the BBB, as microglia contribute to the closure of small openings in the BBB produced during the normal replacement of endothelial cells or in pathological situations (Lou et al., 2016; Taylor et al., 2018). Hence, the absence of microglia would result in more openings in the BBB and increased access to the nervous parenchyma.

Recently, it has been shown that activated microglia probably interfere with entry, proliferation, survival, and/or migration of infiltrating macrophages at the level of the spinal cord, as depletion of microglia results in a larger proportion of macrophages at the lesion and their spreading to other regions of the nervous parenchyma (Plemel et al., 2020). The authors propose that microglia could affect the infiltration of macrophages either directly by secreting factors affecting the macrophage dynamics (as IGF-1), or indirectly by protecting the extracellular matrix (for example microglial cells produce protease inhibitors) and/or by protecting and repairing the BBB (as described above).

From all those studies, it emerges that the entry of myeloid cells to the nervous parenchyma is related to a functional alteration of the BBB. Some of the myeloid cells that enter would give rise to long-lasting MLC depending on the presence of resident microglia. In the absence of microglia, i.e., in an “empty microglial niche”, part of the myeloid cells would give rise to MLC. Once in the CNS, MLC would proliferate and maintain as an autonomous population, as described in section “Maintenance of the Microglial Population” for original microglia (Figure 3B). However, the engraftment of BM-derived cells, when the microglial niche is not occupied and the BBB is apparently unaltered, opens the question of the relative importance and interrelation between these two factors.

## Development of The Microglia/Microglia-Like Identity

Although microglia are supposed to be the tissue-resident macrophages of the nervous parenchyma, they are considered apart from other macrophages because of their origin and maintenance. Microglial cells are assumed to originate and populate the nervous tissue before the closure of BBB that isolates them from the rest of the embryo at a time when only YS precursors are available. In contrast, most other tissue-resident macrophages derive from fetal monocytes, while in some organs, like the dermis and gut, they are continuously replaced by monocytes derived from BM (Sheng et al., 2015; Marquez-Ropero et al., 2020).

Interestingly, transplantation of different types of macrophages (from YS, from fetal liver, or from mature monocytes) reconstitutes alveolar macrophages in mice lacking this population, suggesting that the precursors adopt the features of the macrophage-resident cells of the environment where they were located (van de Laar et al., 2016). Similar observations lead to the assumption that signals issued from the local environment would shape the immunophenotypic and functional features of resident macrophages (Guilliams and Scott, 2017; Bleriot et al., 2020; Guilliams et al., 2020).

Nevertheless, the case of microglia is apparently different, as “true microglial cells” are only produced from YS progenitors, whereas BM-derived progenitors give rise to MLC, which do not adopt a complete microglial identity in a neural microenvironment (Bruttger et al., 2015; Bennett et al., 2018; Cronk et al., 2018; Shemer et al., 2018). Accordingly, BM cells engrafting the CNS undergo morphological (e.g., they adopt a morphology like the original microglia and acquire radioresistance) and molecular modifications (e.g., they partially adopt the epigenetic landscape of microglia). They share around 90% of their transcriptome with host microglia, including the expression of some key microglial genes such as *Tgbr2*, encoding the receptor of TGF-β. However, they remain transcriptionally different from original microglia, as they show a reduced expression of some mRNAs highly expressed in mature microglia, such as *Tmem 119* and *P2yr12*, and do not express the transcriptional regulator *Sall1* (Shemer et al., 2018).

### Environmental and Intrinsic Traits Shaping the Microglia/Microglia-Like Identity

Early work has already revealed the importance of the environment for the acquisition of microglial features: Isolated microglia cultured on an astrocyte layer (to mimic a neural environment) adopt a ramified morphology in contrast to the amoeboid morphology shown by microglial cells in standard culture conditions (Sievers et al., 1994). More recent observations corroborate that factors released by astrocytes promoted the survival and morphological differentiation of microglial cells (Schilling et al., 2001; Bohlen et al., 2017). On the contrary, isolated microglia cultured in a “non-neural” medium reduce the expression of microglial signature genes (as *Tmem119* and *P2ry12*), although they recover their entire microglial identity if transplanted to a brain lacking microglia (Bohlen et al., 2017). *in vivo* experiments also support the relevance of the neural environment for the acquisition of the “microglial identity”: Isolated microglial cells of YS origin transplanted to the brain of a *Csf1r^−/−^* mouse (lacking microglia) produced true microglia (Bennett et al., 2018).

Evidently, if the neural environment were the only factor determining the development of microglia features, it could be expected that any precursor placed in the brain would become microglia. However, the aforementioned authors (Bennett et al., 2018) report that BM-derived cells transplanted into brains lacking microglia originate MLC instead of giving rise to true microglia, as they do not express key signature genes of true microglia, like *Sall1*. Comparable results have been reported elsewhere (Cronk et al., 2018; Lund et al., 2018; Shemer et al., 2018), indicating that the ability to give rise to “true microglia” seems to be restricted to YS-derived precursors. Indeed, YS-derived embryonic macrophages (EM) and BM-derived macrophages (BMDM) co-cultured with neural stem/progenitor cells show clear differences. While EM differentiates to microglial cells which express *Tmem 119* mRNA, a marker for microglia with advanced or total differentiation (Bennett et al., 2016), BMDM cultured in the same conditions do not express *Tmem 119* at noticeable levels, although they adopt a microglia-like morphology and are positive for Iba-1 (Yosef et al., 2018). Therefore, the acquisition of the microglial identity depends on both cell intrinsic and environmental factors (Bennett et al., 2018).

Apparently, the embryonic experience of YS precursors shapes the features of true microglia required for the CNS development, while MLC precursors, that act when the CNS is already developed, do not maintain these features, and lose some of the characteristics of true microglia. Many of the “signature genes” of YS-derived microglia are regulated by the transcription factor Sall1 (Guilliams et al., 2020), and, therefore, the expression of the *Sall1* gene is likely to play a key role in establishing the microglial identity (Buttgereit et al., 2016; Lund et al., 2018; Shemer et al., 2018). Different hypotheses have been proposed to explain why MLC derived from BM do not express key microglial genes, even though the neural environment induces the expression of other “microglial” genes. Thus, it has been suggested that chromatin accessibility in BM-derived cells precludes the expression of *Sall1* or that a specific signal in the embryonic brain is needed to induce *Sall1* expression (Guilliams et al., 2020).

A recent report (Chen et al., 2020) reveals that fetal monocytes enter the brain parenchyma during development and after a neonatal stroke. Once in the parenchyma, they progressively adopt a microglial identity, expressing microglial signature genes including *Sall1*. The authors conclude that fetal CCR2+ monocytes (originated from fetal liver hematopoiesis) might contribute to true microglia in the mouse brain. Therefore, the ability to originate true microglial cells would not be exclusive to cells produced directly in the YS. Rather this capacity could progressively be lost during the development of the hematopoietic system, as fetal monocytes, but not adult ones, maintain it.

The identity of the precursors of true microglia has been already discussed in section “Microglial Origin From the Yolk Sac”. MLC may derive either from immature hematopoietic precursors or from circulating monocytes. In relationship with the monocyte origin, at least two monocyte populations have been described in the circulating blood of mice: Ly-6C^low^ (CCR2^−^ and CX3CR1^hi^) and Ly-6C^hi^ (CCR2^+^ and CX3CR1^low^). The latter one is thought to originate MLC (Mildner et al., 2007; Lund et al., 2018).

These results do not discard the possibility that BM-derived HSC would be involved in the production of MLC. Although low numbers of HSC are present in the circulating blood in healthy conditions (Schreier and Triampo, 2020), inflammatory stimuli elicit the increased mobilization of HSC from the BM to the blood (Ratajczak et al., 2018). Thus, the increased number of engrafted myeloid cells observed in the diseased brain might relate to the higher number of immature hematopoietic precursors present in the peripheral blood in pathological conditions. In fact, a study claims that the ability to generate MLC is restricted to uncommitted HSC or progenitor cells from the BM (Ajami et al., 2011).

Highlighting the relevance of hematopoietic precursors in the production of MLC, it has been reported that they replace around 92% of the original microglia in the brain when BM cells are transplanted to irradiated and microglia-depleted mice. However, this rate is reduced to around 80% after peripheral blood transplantation (Xu et al., 2020). It is likely that more HSC are present in the BM transplant than in the peripheral blood transplant, and this could result in a larger rate of replacement. Associated with that is the surprising observation that transplantation of a single HSC from BM gives rise to a comparable engraftment of myeloid cells in the CNS as transplanting 10^6^ cells from the whole BM (Massengale et al., 2005).

In summary, the acquisition of a microglial identity depends on different factors. One of them is the neural environment; the other one is the intrinsic characteristics of the cells, as only hematopoietic cells from the embryo seem to be able to produce true microglia, whereas adult hematopoietic cells originate MLC.

### Functional Differences Between Microglia and MLC

Despite that MLC resemble microglia in morphology and in the expression of many markers, they respond to injury differently (Bennett et al., 2018). Although both cell types actively move toward injured areas of the brain, MLC move faster towards a laser burn than microglia and MLC do not change their ramification in response to an intraperitoneal LPS injection, while microglia do. In addition, many genes were differentially expressed in microglia and MLC after LPS treatment. For example, transcripts encoding the scavenger receptor Marco are induced in microglia but not in engrafted cells (Cronk et al., 2018; Shemer et al., 2018), indicating that microglia of embryonic origin and MLC are not totally equivalent. In fact, BM-derived cells seem to be more efficient than YS-derived microglia in particular tasks, mostly associated with aging, such as phagocytosis of amyloid deposits or debris generated during senescence (Shemer et al., 2018; Li et al., 2020). In this sense, it is well known that both microglia and macrophages of blood origin fulfill different roles after a brain injury (Amici et al., 2017; Mesquida-Veny et al., 2021). However, to our knowledge, the functional differences between microglia and MLC are not well established, for example, if they have similar functions in parenchyma surveillance, sculpting of nerve projections, and synaptic pruning.

There are reports suggesting that microglia and BM macrophages are very similar. During the acute phase of EAE, microglia downregulate some of the microglial-specific genes (Butovsky et al., 2014; Jordao et al., 2019; Grassivaro et al., 2020), resulting in RNA levels similar to infiltrating macrophages in the same phase. Additionally, mRNA expression of some of the microglial genes is transiently enhanced in macrophages of BM origin during the chronic phase of EAE (Grassivaro et al., 2020). This has led the authors to propose that microglia and macrophages of BM origin phenotypically converge after a brain injury to act as a single functional unit and that therefore both cell types might not be contemplated as totally different entities (Grassivaro et al., 2020).

In any case, BM cells invading the nervous parenchyma look and act like microglia, although they cannot be considered as true or “*bona fide*” microglia (McPherson et al., 2019). For example, no behavioral effects are observed in mice with a significant replacement of microglia by engrafted cells of BM origin (Cronk et al., 2018), suggesting that the level of microglia replacement does not affect brain function.

## Human vs. Mouse Microglia

The former discussion has focused on microglia and MLC in the murine brain. However, for therapeutic purposes, we need to increase our knowledge of microglia and MLC in the human brain. Several studies have dealt with human microglia and their similarities to mouse microglia (Menassa and Gomez-Nicola, 2018; Masuda et al., 2020; Zia et al., 2020). A gene expression study of microglia in 10 different species (Geirsdottir et al., 2019) reveals that human microglia show a larger heterogeneity than murine ones. This can be related to the fact that more pathological events occur in the human brain than in the brain of other species because of their longer life span. These events presumably produce the development of particular subsets of microglia (Keren-Shaul et al., 2017; Masuda et al., 2020) and more frequent myeloid cell engraftment. Therefore, an appreciable number of MLC is supposed to be present in the brain of long-lived organisms, such as humans, because of the repeated production of pathological events (often with only subclinical effects) that are frequently associated with local disruption of BBB and limited depletion of microglia.

Recently, it has been reported that the stromal fraction of human BM contains a CD45^−^ CD11b^+^ precursor, apparently devoted to producing cells labeled with Iba-1 and expressing genes of developing (as *RUNX1*, *SPF1*, and *CSF1R*) and adult human microglia (including *P2RY12*, *TMEM119*, and *ITGAM*). Moreover, cells produced from these precursors show increased phagocytic capacity, are activated in response to LPS, and integrate into human brain tissue, conserving the expression of TMEM119 marker (Bruzelius et al., 2021). Therefore, it seems that human BM cells might be able to give rise to new microglia. To our knowledge, a similar precursor in the mouse BM has not been reported until now.

## Discussion and Conclusions

While the original microglia in mammals mostly derive from non-replaced embryonic precursors of YS origin, the first macrophages and/or microglia appearing during development in zebrafish and chicken are replaced by cells derived from intraembryonic or definitive hematopoiesis (Garceau et al., 2015; Ferrero et al., 2021). The persistence of the original microglia in mammals may reflect a greater isolation of the nervous parenchyma precluding the entry of new microglial precursors. This would account for differences in microglia between mammals and other groups. In fact, analysis of the microglial gene expression reveals that zebrafish and chicken microglia show an expression pattern that situates them out of the mammalian group (Geirsdottir et al., 2019).

In contrast to homeostatic conditions, different events affecting the CNS allow large engraftment of BM-derived cells in the brain parenchyma. This change is apparently related to the presence of an empty microglial niche and the accessibility of precursors to the parenchyma because of the alteration of BBB permeability. The acquisition of microglial features might depend on: (a) intrinsic properties of the precursors because only cells deriving from the YS give rise to “true microglial cells”, while BM-derived cells are only able to give rise to MLC; and (b) local cues, as conditions mimicking a nervous system environment—for example co-cultures with astrocytes or neurons, astrocyte conditioned medium—are needed to acquire microglial (or MLC) characteristics.

Therefore, the microglial compartment of the mammalian adult brain integrates two different components: “true microglial cells” and MLC. Whereas the first one is of embryonic origin, MLC are myeloid cells of BM origin that engraft the brain in relationship with the depletion of the original microglia and/or when the BBB is compromised. Consequently, MLC likely integrate into the original network of microglia of embryonic origin and could carry out similar functions.

The topic discussed in this review is relevant to the development of therapeutic strategies in diseases in which microglial cells are involved (Bennett and Bennett, 2020; Xu et al., 2020), such as neurological/psychiatric disorders like autism spectrum disorders and compulsive behavior, ischemia, neurodegeneration, and traumatic injury. There is strong evidence that original microglia of embryonic origin can be replaced by myeloid cells of BM origin in the human brain, similar to the findings in the mouse CNS (Menassa and Gomez-Nicola, 2018; Zia et al., 2020). This can represent a scientific basis to use myeloid transplants to treat CNS diseases with defective microglia, as neuropathologies developing in aged people when microglia is dysfunctional (Ng et al., 2021), or some forms of leukoencephalopathies in which CSFR-1 is deficient and microglial phenotype is altered (Kempthorne et al., 2020). In these cases, transplants would replace toxic or noxious microglia with healthy cells able to restore homeostasis. However, extensive depletion of the microglial niche might be necessary to replace these defective microglia. Thus, future experiments have to establish optimal conditions and procedures to achieve this goal.

## Author Contributions

MAC designed the manuscript, wrote the first draft and participated in editing. MRS, DM-O, and JLM-T contributed to the editing and writing of the final version. VEN edited the manuscript and prepared the figures. All authors contributed to the article and approved the submitted version.

## Conflict of Interest

The authors declare that the research was conducted in the absence of any commercial or financial relationships that could be construed as a potential conflict of interest.

## Publisher’s Note

All claims expressed in this article are solely those of the authors and do not necessarily represent those of their affiliated organizations, or those of the publisher, the editors and the reviewers. Any product that may be evaluated in this article, or claim that may be made by its manufacturer, is not guaranteed or endorsed by the publisher.

## Abbreviations

4’OHT: hydroxytamoxifen
AGM: aorta-gonad-mesonephros
BAM: border-associated macrophages
BBB: blood-brain barrier
BM: bone marrow
BMDM: bone marrow-derived macrophages
CNS: central nervous system
CSF-1: colony stimulating factor-1
DTR: diphtheria toxin receptor
EAE: experimental autoimmune encephalitis
EM: embryonic macrophages
EMP: erythromyeloid progenitor
FIRE: *fms*-intronic regulatory element
GFP: green fluorescent protein
HSC: hematopoietic stem cells
HVTK: herpesvirus thymidine kinase
IL-34: interleukin-34
LPS: lipopolysaccharide
MLC: microglia-like cells
PBI: posterior blood islands
PLM: posterior lateral mesoderm
RBI: rostral blood islands
TGF-β: transforming growth factor-β
VDA: ventral wall of dorsal aorta
YS: yolk sac.
